# Expression of cyclin-dependent kinases and their clinical significance with immune infiltrates could predict prognosis in colorectal cancer

**DOI:** 10.1016/j.btre.2021.e00602

**Published:** 2021-02-23

**Authors:** Adewale Oluwaseun Fadaka, Nicole Remaliah Samantha Sibuyi, Olalekan Olanrewaju Bakare, Ashwil Klein, Abram Madimabe Madiehe, Mervin Meyer

**Affiliations:** a Department of Science and Innovation/Mintek Nanotechnology Innovation Centre, Biolabels Node, Department of Biotechnology, Faculty of Natural Sciences, University of the Western Cape, Bellville, South Africa; b Bioinformatics Research Group, Department of Biotechnology, Faculty of Natural Sciences, University of the Western Cape, Private Bag X17, Bellville, 7535, Cape Town, South Africa; c Plant Omics Laboratory, Department of Biotechnology, Faculty of Natural Sciences, University of the Western Cape, Private Bag X17, Bellville, 7535, Cape Town, South Africa; d Nanobiotechnology Research Group, Department of Biotechnology, Faculty of Natural Sciences, University of the Western Cape, Bellville, South Africa

**Keywords:** Cyclin-dependent kinases, Prognostic value, Colorectal cancer, Biomarkers, Expression, Immune infiltrates

## Abstract

**Introduction:**

Colorectal cancer (CRC) is one of the most cancer-related mortalities worldwide and remains a major public health issue. Despite several attempts to develop promising therapies for CRC, its survival rate decreases with metastasis. Cyclin-dependent kinases (CDKs) are a family of protein kinases with various regulatory activities including cell cycle, mRNA expression, transcription, and differentiation. Aside from their role in cell proliferation when mutated, abnormal expression of these genes has been reported in some human cancer subtypes. This study explored the roles and therapeutic potentials of CDK 1 and 4 as prognostic biomarkers in CRC.

**Methods:**

Bioinformatics analyses were carried out to demonstrate the expression and prognostic values of CDK-1 and CDK-4 with immune infiltrate in CRC.

**Discussion:**

CDK levels in CRC were remarkably higher than those in normal tissues (*p* < 0.05), and overexpression in CRC tissues was significantly related to nodal metastatic status (*p* < 0.05) and histological subtypes. Kaplan-Meier analyses showed that patients with CRC who exhibited CDK-1 overexpression had worse overall survival (OS) as against patients with CDK-4 overexpression. The alteration observed was a mutation while the mutation hotspots include E163* and R24A/C/H/L respectively for CDK-1 and CDK-4 on the Pkinase domain. Of the associated genes, AURKA and RB1 were predominantly altered. Furthermore, CDK-4 is positively correlated with tumor purity in both COAD and READ while CDK-1is only positively correlated in COAD. CDK-1 overexpression was significantly associated with poor prognosis as opposed to CDK-4.

**Conclusion:**

The expression and prognostic values of AURKA and RB1 may also be significant to CRC diagnosis. CDKs together with the co-expressed genes and their association with immune infiltrates may serve as target molecules for immunotherapy in CRC.

## Highlights

• The expression and prognostic values of AURKA and RB1 may also be significant to CRC diagnosis than previously studies.
• The association of CDKs with immune infiltrates may serve as target molecules for immunotherapy in CRC.
• The expression of CDK is significant among CRC subtypes and therefore, it can be inferred as a potential biomarker in the cancer subtype.
• An increase in tumor purity was positively correlated with the expression of CDK-1 in COAD due to CD4+ cells and CDK-4 in COAD and READ resulting from a fraction of immune cells.

## 1 Introduction

Colorectal carcinogenesis is a sequential process that results from genetic alterations in either oncogenes or tumor-suppressor genes. Molecular alterations of genes have also been implicated in the development and prognosis of CRC. The survival rate of CRC patients relies on the stage during diagnosis and decreases as the tumor advances [1]. To date, the mechanisms that drive the pathogenesis of CRC are poorly understood and the development of resistance to cytotoxic and targeted therapies remains the primary reason for treatment failure. Therefore, the development of new therapeutic targets that can translate into better clinical outcomes is urgently required for individuals with CRC. Additionally, the identification of biomarkers for early diagnosis and prognosis could improve the efficiency of current treatments for CRC and represent molecular markers for therapeutic targets.

**Fig. 1:**
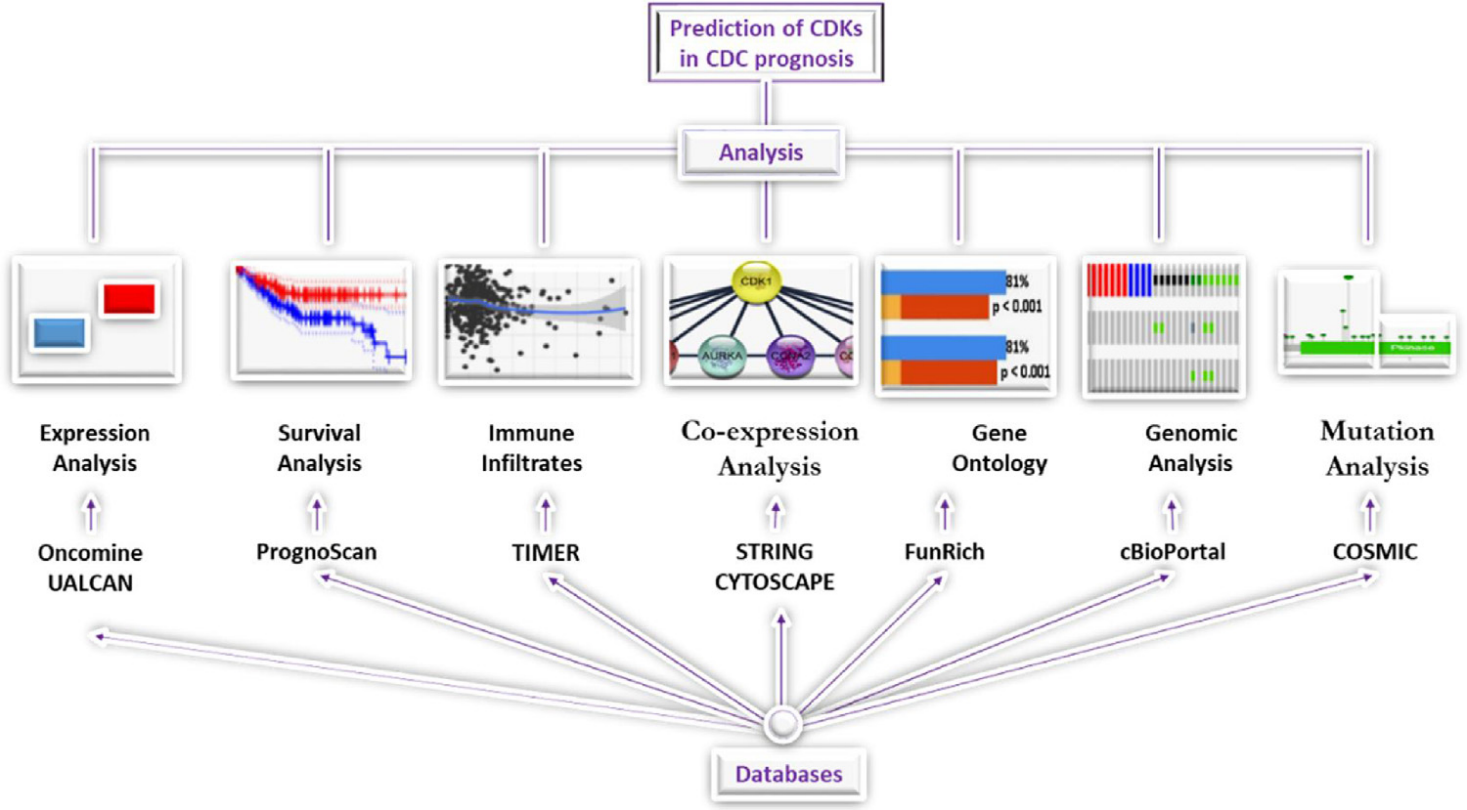
Schematic representation of the workflow.

Cyclin-dependent kinases (CDKs) are such potential biomarkers for the diagnosis of CRC due to their crucial role in the elongation step of the global transcription process [2]. CDKs and their specific functions in cancer have been comprehensively investigated; they are involved in regulation of the cell cycle by interacting with specific cell-cycle–regulatory cyclins [3]. The CDK family consists of 20 members (CDK-1 to CDK-20). This class of serine/threonine kinases has been classified into two groups based on their involvement in tumor formation through the regulation of cell-cycle or transcription [[4], [5], [6]]. CDK-1, CDK-4, and CDK-5 fall within the cell-cycle-related subfamilies while CDK-7, CDK-8, CDK-9, CDK-11, and CDK-20 are classified as transcriptional subfamilies. Malumbres and Barbacid [7] reported that mammalian cells require a step-wise activation of at least four different CDKs to drive cells through interphase, and for CDK-1 to proceed through mitosis. CDK-1 promotes the G_2_–M transition and regulates G_1_ progression and G_1_–S transition [8,9], suggesting that CDK-1 is one of the most important CDKs for cell-cycle regulation [10]. Uncontrollable cell proliferation is one of the hallmarks of malignant tumors and is often driven by mutations in CDK activity. Altered CDK expression and/or activity is observed in many cancer subtypes [11,12]. In addition to cell-cycle regulation, CDK-1 may regulate apoptosis by inhibiting phosphorylation of caspase-8 [13,14]. CDK-1 functions include apoptosis, senescence, angiogenesis, cell cycle progression, and adhesion/migration [[15], [16], [17]], all of which are believed to contribute to its role in tumorigenesis. CDK-1 has been implicated in several cancers, including ovarian [18,19], thyroid [20], bladder [21], colorectum [22], breast [23,24], and liver cancer [25]. CDK-4 is also a key player in cell cycle and mediates cell progression through the G1 phase [26]. Its dysregulation has been found to play a significant role as regulator of signaling in cellular pathways in several diseases. Since the frequent deregulation of CDK-4 in cancer often leads to addiction to its activity, it is emerging as a bright therapeutic target [27,28]. CDK inhibitors were recently investigated in preclinical and clinical trials. These inhibitors are believed to act as anti-cancer drugs by blocking CDKs to prevent uncontrolled cellular proliferation that is a hallmark of cancers specifically in CRC [29]. In earlier studies, the relationship between genetic mutations and CDK overexpression were investigated [30]. The overexpression of CDK4 was observed in colorectal tumors that carry the type II transforming growth factor-β receptor mutation [31]. This mutation resulted in microsatellite instability in 15 % CRC patients [31]. Zhao et al. [32] reported the expression of CDK-4 in 74 specimens through grading, and concluded that stronger immunostaining for CDK-4 was predictive of a worse prognosis (P < 0.001). Tumor immune cells play essential roles in cancer development and progression. However, the association with prognosis in CRC remains elusive [33]. A recent study showed that the expression of CDK-9 was negatively associated with the infiltration of CD8^+^ T cells at the tumor site in CRC patients [34]. Thus, CDKs may have roles in tumorigenesis, progression, and metastasis, and may serve as an underlying prognostic factor for tumors. Since the TCGA transcriptomic datasets have proven the relevance of CDKs in CRC [35], targeting CDKs could prove an effective strategy to enhance the efficacy of treatment in CRC. With clear emphasis on CDK-1 and CDK-4, the association between CDKs expression and clinical pathological parameters, including prognostic value, was investigated, and the biological function and immune infiltrate activities of CDKs in patients with CRC was examined using an *in silico* approach (Fig. 1). This study suggests that the involvement of CDKs in CRC has a pivotal clinical significance in CRC.

## 2 Materials and methods

### 2.1 mRNA expression analysis

The web-based Oncomine database accessed at https://www.oncomine.org/resource/login.html, was used to investigate the transcription levels of CDK-1 and CDK-4 in CRC using the following parameters: *p*<1 × 10^‑4^, fold-change >2 and gene ranking in the top 10 %. The mRNA expressions were compared between normal and tumor clinical samples.

### 2.2 Prognostic analysis

The correlation between CDK-1 expression and survival in CRC was analyzed by PrognoScan database (http://www.abren.net/PrognoScan/), Gene Expression Profiling Interactive Analysis (GEPIA) (http://gepia.cancer-pku.cn/index.html) and UCSC-Xena (https://xena.ucsc.edu/kaplan-survival-analysis/). Xena analyzes cancer genomics and its associated clinical data. The threshold was adjusted to cox *p*-value < 0.05. The PrognoScan database was used to validate the prognostic value of CDK-1 and CDK-4 expression in patients with CRC. The adjusted *p*-value threshold was <0.05.

### 2.3 UALCAN database analysis

UALCAN is a comprehensive, user-friendly, and interactive web resource for analyzing cancer OMICS data [36]. The correlation between CDKs expression and lymph node metastasis in colon and rectum adenocarcinomas was analyzed using UALCAN (http://ualcan.path.uab.edu/). CDK-1 expression and the clinicopathological parameters relationship was provided in a tabular form.

### 2.4 Predicted protein interaction

Co-expressed proteins were identified according to a method previously described by Fadaka et al. [37]. The database was accessed, downloaded and installed from http://www.cytoscape.org/ (v3.7.2) along with the STRING plug-in (http://string-db.org/ v11). Only genes with a 0.90 confidence level as the minimum required interaction score were considered.

### 2.5 Gene ontology

FunRich is a stand-alone software tool used mainly for functional enrichment and interaction network analysis of genes and proteins [38]. The enrichment analyses of gene ontology such as cellular component (CC), biological processes (BP), molecular function (MF) and biological pathway were analyzed using the functional enrichment (v3.1.3) software accessed at http://funrich.org/index.html.

### 2.6 Mutations analysis for CDK-1 and CDK-4

The Catalogue of Somatic Mutations in Cancer (COSMIC) database coupled with cBioPortal was used to detect genetic mutation frequencies and hotspots for CDK-1 and CDK-4. The COSMIC v90, (released 05-SEP-19) database accessed at https://cancer.sanger.ac.uk/cosmic is a comprehensive resource for exploring the effect of somatic mutations in all human cancer subtypes [39,40]. This database also provides information pictorially on the distribution and substitutions on the coding strand in cancer subtypes.

### 2.7 Genomic analysis of CDK-1 and CDK-4

Cancer genomics analysis was performed using the cBioPortal database for Cancer Genomics (http://www.cbioportal.org/). This tool was used to investigate the genes positively associated with CDK-1 and CDK-4 expression in CRC and the RNA sequencing data and copy number variance. cBioPortal was also used to analyze the alteration frequency of CDK-1 and CDK-4 mutations in CRC. The ten combined studies used are listed as follows: Colon Cancer (CPTAC-2 Prospective, Cell 2019) [41]; Colorectal Adenocarcinoma (TCGA PanCancer Atlas) [42]; Colorectal Adenocarcinoma (TCGA Firehose Legacy); Colorectal Adenocarcinoma (TCGA, Nature 2012) [43]; Colorectal Adenocarcinoma (Genentech, Nature 2012) [44]; Colorectal Adenocarcinoma (DFCI, Cell Reports 2016) [45]; Metastatic Colorectal Cancer (MSKCC, Cancer Cell 2018) [46]; Rectal Cancer (MSK Nature Medicine 2019); Colon Adenocarcinoma (CaseCCC, PNAS 2015) [47]; Colon Adenocarcinoma Triplets (MSKCC, Genome Biol 2014) [48].

### 2.8 Immune Cells Tumor-Infiltration analysis

Tumor Immune Estimation Resource (TIMER v 2.0), a web-based database for comprehensive analysis of tumor-infiltrating immune cells [49,50] (https://cistrome.shinyapps.io/timer/) was used to assessed the interactions between the genes of interest and the immune system for better understanding of CDKs-immune interactions in CRC. Six immune infiltrates namely; B cells, CD4^+^ T cells, CD8^+^ T cells, Neutrophils, Macrophages and Dendritic cells were analyzed using this algorithm. Furthermore, the expression report of Oncomine was validated by TIMER v2.0.

### 2.9 Statistical analysis

Based on the Bioinformatics approaches employed, the Kaplan-Meier plot was generated by PrognoScan, GEPIA, and UCSC-Xena. PrognoScan and GEPIA analyses were displayed along with Hazzard ratio (HR) and *p*-values of the log-rank test. Expression analysis results were presented as fold changes, *p*-values, and ranks. The difference in expression was verified using Student’s *t*-test. Differences between and within groups were carried out using ANOVA for multiple tissues. The correlation of gene expression was assessed by statistical significance, Pearson's correlation, and spearman’s correlation. The scatter/ boxplot was created using TIMER computational tool and was calculated using spearman’s correlation between two groups of genes of interest. The significance level was set as *p* < 0.05.

## 3 Results

### 3.1 Expression of CDKs

**Fig. 2:**
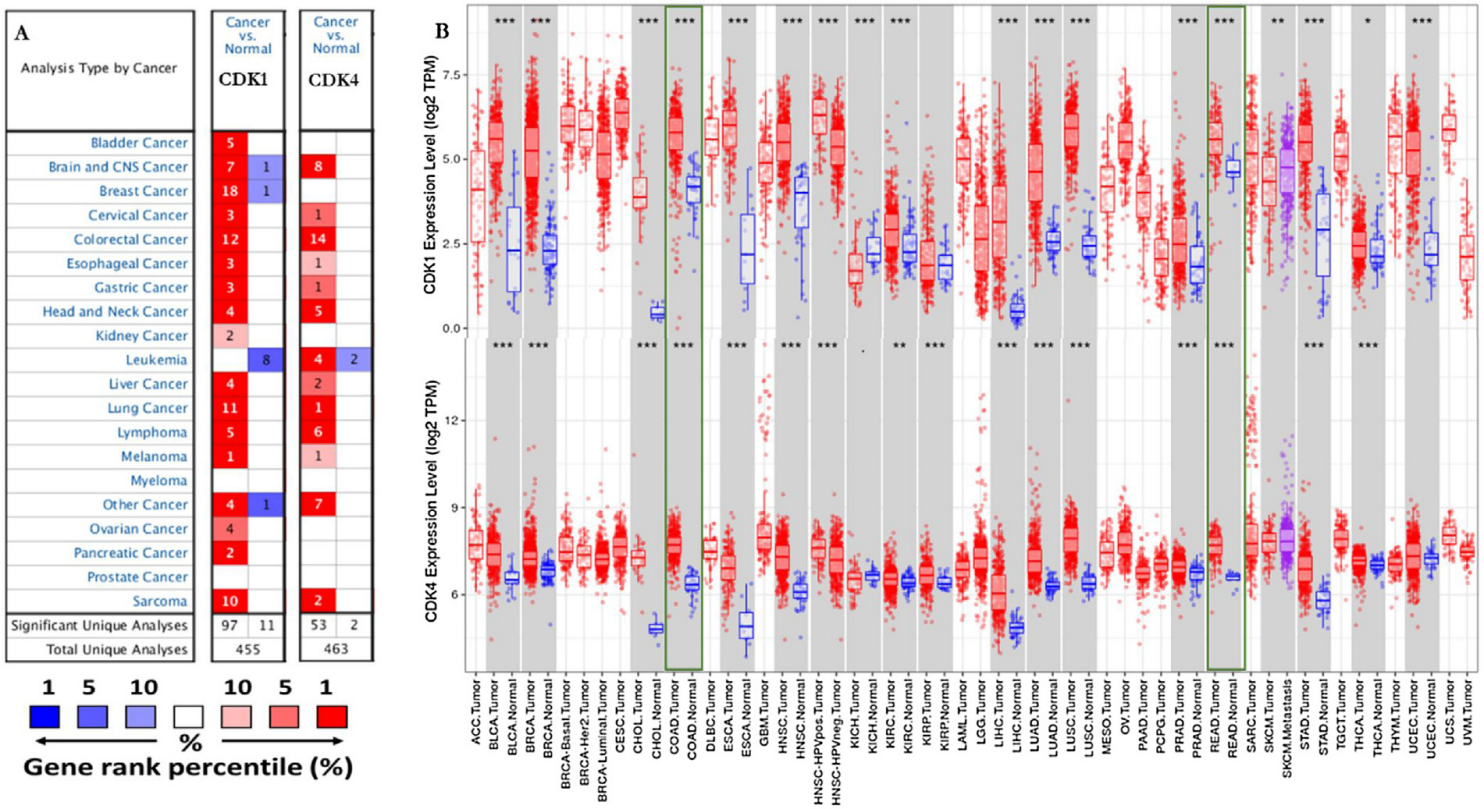
mRNA expression of CDK-1 and CDK-4 in various cancer types. The mRNA expression of CDK-1 and CDK-4 (cancer against normal tissue) was evaluated using the Oncomine (A) and TIMER (B) computational tools. The red color represents significant overexpression whereas blue color represents reduced expression. The green box highlights the cancer of interest, *p < 0.05, **p < 0.01 and ***p < 0.001.

**Fig. 3:**
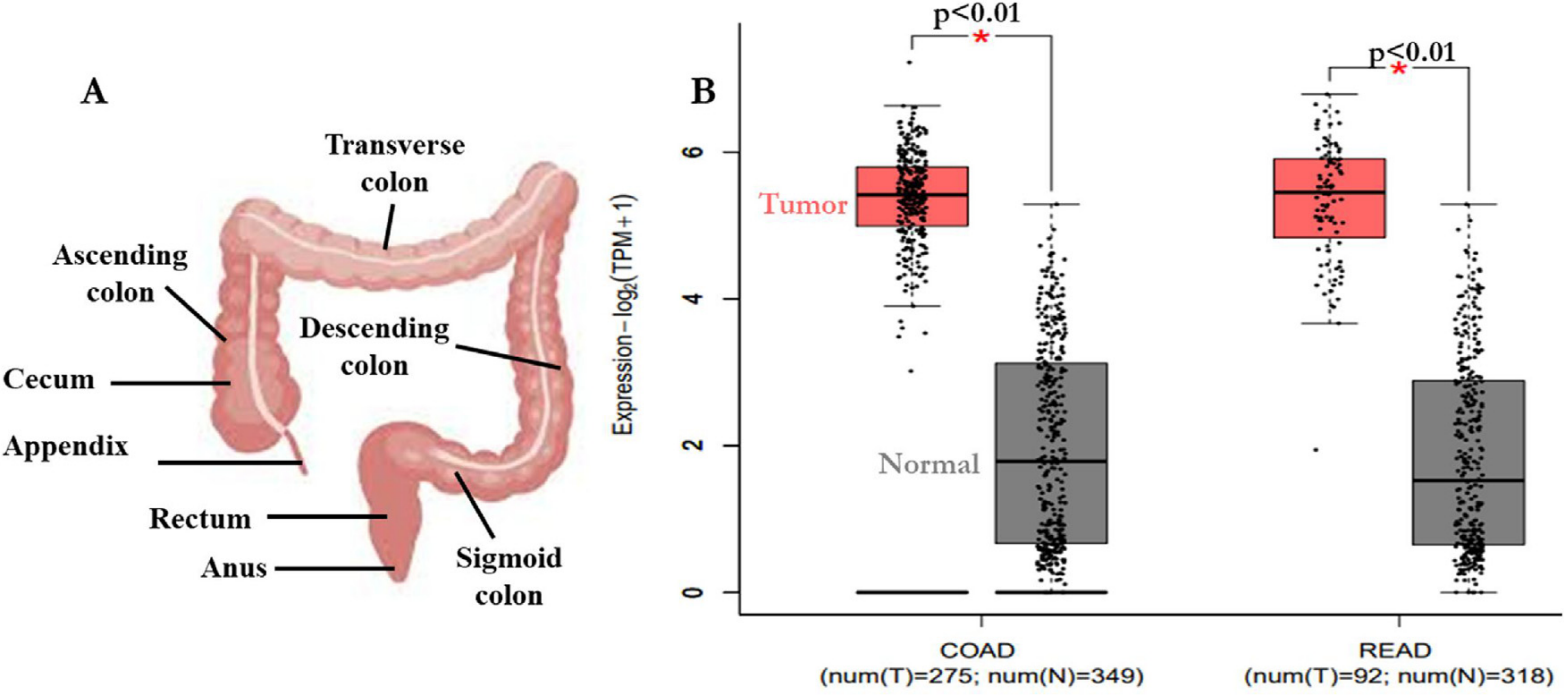
Anatomy of the colorectum and expression signature of CDK-1 and CDK-4 in colon (COAD) and rectum (READ) adenocarcinomas. The different sections (labeled) of the colorectum (A) can be affected by CRC. Expression signatures of CDK-1 and CDK-4 in COAD and READ was analyzed using the GEPIA database (B).

To determine the expression of CDKs among subtypes of CRC, it is important to explore their expression among different cancer types (Figs. 2a, b and 3 ). The expression of these genes was investigated in both solid tumors as well as hematological malignancies (Fig. 2). The expression of CDK-1 was upregulated in 17 cancers including breast cancer, CRC, lung cancer and sarcoma in total unique analyses of 455 different datasets. Underexpression of this gene was also identified in brain and central nervous system (CNS) cancer, breast cancer and leukemia. Although the expression of CDK-4 was determined in 463 different analyses, its expression was up-regulated in 53 cancer datasets and downregulated in two cancer studies (leukemia). The pathogenesis of CRC is unique among human tumors. This common malignant tumor usually develops in a benign precursor lesion, the adenoma, which is visible on the mucosal surface of the large bowel, constituting an adenoma-adenocarcinoma sequence. The expression of CDK-1 and CDK-4 were significantly elevated in multiple CRC types when compared to normal colorectum tissues (*p* = 1.0 × 10^−4^). These CRC types include Colon Adenocarcinoma, Cecum Adenocarcinoma, Cecum Adenocarcinoma, Rectosigmoid Adenocarcinoma, Colon Mucinous Adenocarcinoma, Rectal Mucinous Adenocarcinoma, Rectal Adenocarcinoma, Colon Adenocarcinoma, Colon Adenoma, Rectum Adenoma, Colon Adenocarcinoma, Rectal Adenocarcinoma, Colorectal Carcinoma and Colorectal Adenocarcinoma with different *p*-values and fold changes (Table 1 and Fig. 5).

**Table 1:** CDK-1 mRNA expression in CRC compared to normal tissues (Oncomine).

CRC Subtype	Fold-change	P‑value	Rank (%)	Sample	Source
Colon Adenocarcinoma	2.025	3.73 × 10^−6^	1	62	[51]
Cecum Adenocarcinoma	3.437	6.72 × 10^−19^	1	237	TCGA, 2011
Rectosigmoid Adenocarcinoma	2.059	6.07 × 10^−10^	1	237	TCGA, 2011
Colon Mucinous Adenocarcinoma	3.249	8.81 × 10^−14^	1	237	TCGA, 2011
Rectal Mucinous Adenocarcinoma	3.348	8.89 × 10^−7^	1	237	TCGA Colorectal, 2011
Rectal Adenocarcinoma	3.181	4.15 × 10^−19^	3	237	TCGA Colorectal, 2011
Colon Adenocarcinoma	3.374	3.27 × 10^−18^	4	237	TCGA Colorectal, 2011
Colon Adenoma	2.618	4.10 × 10^−19^	1	64	[52]
Rectum Adenoma	3.027	4.93 × 10^−7^	3	64	[52]
Colon Adenocarcinoma	2.730	1.05 10^−5^	2	36	[53]
Rectal Adenocarcinoma	2.535	1.25 × 10^−35^	1	130	[54]
Colorectal Carcinoma	2.447	4.31 × 10^−12^	1	105	[55]
Colorectal Adenocarcinoma	2.060	1.98 × 10^−10^	4	105	[55]
Colorectal Carcinoma	3.636	8.57 × 10^−11^	4	82	[56]

Note: Adenoma is a benign for of adenocarcinoma. Sometimes this counterpart transforms into adenocarcinomas.

These expressions were further validated using the computational tool (TIMER v 2.0) (Fig. 2b). Clear emphasis were placed on CRC as the cancer type of interest and their expressions were evaluated using the GEPIA database (Fig. 3b).

### 3.2 Genetic alterations of CDKs and clinicopathological parameters in CRC patients

**Fig. 4:**
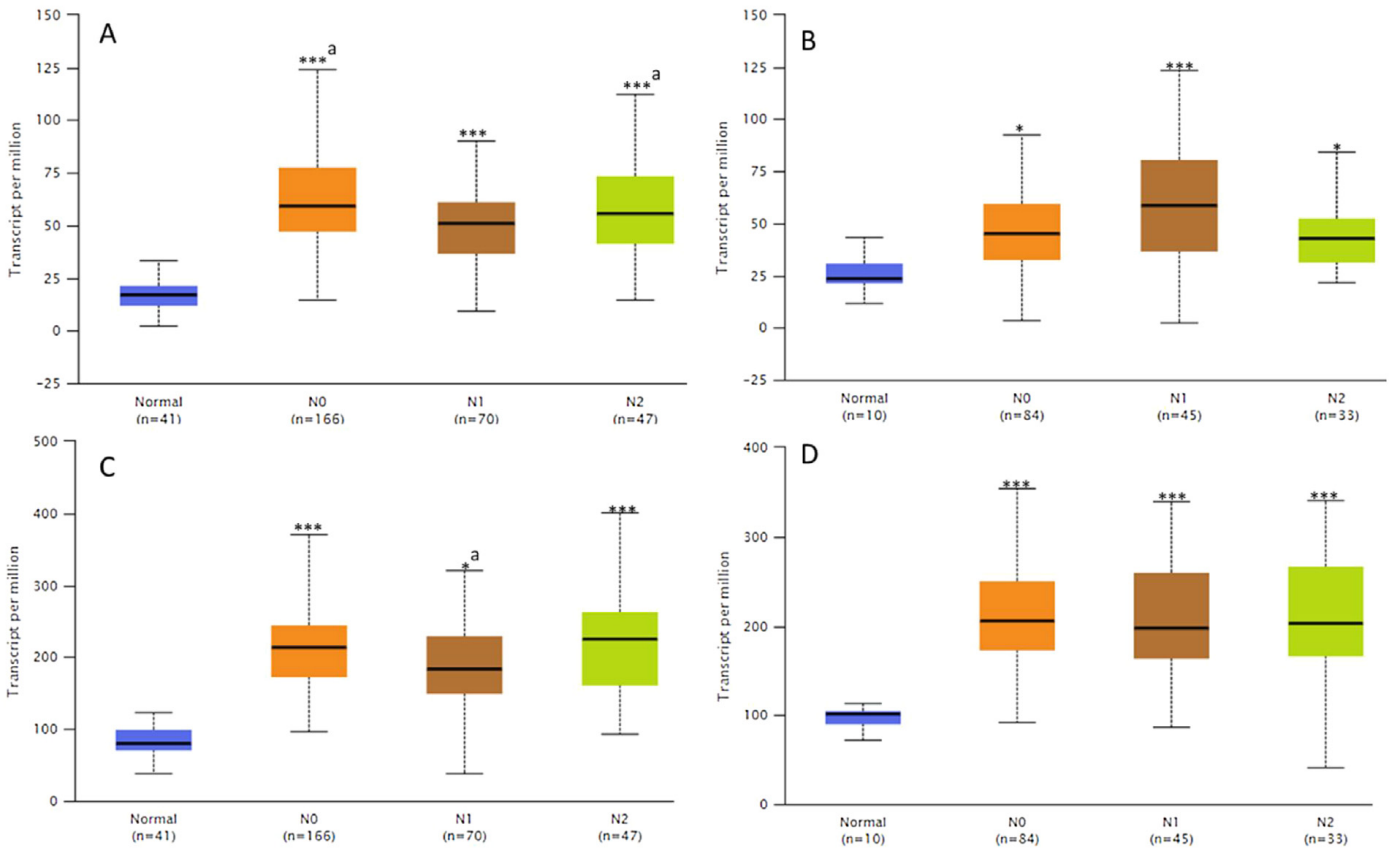
Analysis of CDK-1 and CDK-4 expression in COAD and READ using UALCAN software based on nodal metastasis status. The expression of CDK-1 (A, B) and CDK-4 (C, D) was significantly higher in COAD (A, C) and READ (B, D) tissues with lymph node metastasis than in normal tissues.*p < 0.01, ***p < 0.0001.

To understand the importance and mechanism underlying the expression of CDKs in CRC, the expression profiles of CDKs were examined across CRC subtypes using OMICS data (obtained from the UALCAN database) based on different clinical-pathological parameters (Table 2). This analysis differs from Oncomine expression analysis in Table 1 which evaluated the expression of CRC subtypes compared to normal counterpart. Table 2 specifically investigated normal expression of CDK 1 and 4 in selected clinic-pathological parameters. Based on gender, an increase in CDK expression was observed in males and females with CRC when compared to individuals without the disease. The age criterion demonstrated significantly increased expression of CDK genes. mRNA levels in tumors of patients of all ages >20 - 100 years, compared with normal individuals of all ages. In addition, the CDKs mRNA expression was significantly increased in sample types (*p* < 1.0 × 10^−12^), race, weight, tumor stages, histological subtypes, and nodal metastasis status compared with the corresponding normal tissues (Table 2). The nodal metastatic status of these genes was represented with a box plot using the CRC subtypes Colon adenocarcinoma (COAD) and Rectum adenocarcinoma (READ) (Fig. 4).

**Table 2:** Clinico-pathological parameters of CRC patients and CDK-1 and CDK-4 mRNA expression.

Parameter	Characteristics	Number of cases	CDKs mRNA	CDK-1 *p*-value	CDK-4 *p*-value
*Gender*	Normal (male & female)	41	–		
	Male	156	↑	1.62 × 10^−12^*	1.45 × 10^−15^*
	Female	127	↑	1.00 × 10^−12^*	1.30 × 10^−11^*
*Age*	Normal	41	–		
	21−40yrs	12	↑	1.55 × 10^−06^*	1.11 × 10^−07^*
	41−60yrs	90	↑	1.00 × 10^−12^*	1.00 × 10^−02^*
	61−80yrs	149	↑	1.00 × 10^−12^*	1.60 × 10^−05^*
	81−100yrs	32	↑	1.11 × 10^−16^*	1.91 × 10^−07^*
*Sample types*	Normal	41	–		
	Primary tumor	286	↑	1.00 × 10^−12^*	1.55 × 10^−06^*
*Race*	Normal	41	–		
	Caucasian	195	↑	1.00 × 10^−12^*	3.10 × 10^−11^*
	African American	55	↑	1.62 × 10^−12^*	2.32 × 10^−12^*
	Asian	11	↑	8.33 × 10^−05^*	3.44 × 10^−05^*
*Weight*	Normal weight	41	–		
	Extreme weight	70	↑	1.62 × 10^−12^*	2.42 × 10^−14^*
	Obese	74	↑	1.62 × 10^−12^*	3.62 × 10^−14^*
	Extreme obese	56	↑	1.00 × 10^−12^*	4.00 × 10^−15^*
		10	↑	1.02 × 10^−04^*	2.02 × 10^−14^*
*Stages*	Normal I	41	–		
	II	45	↑	1.00 × 10^−12^*	1.11 × 10^−15^*
	III	110	↑	1.63 × 10^−12^*^a^	5.73 × 10^−16^*^a^
	IV	80	↑	1.625 × 10^−12^*^a^	2.65 × 10^−02^*^a^
		39	↑	1.00 × 10^−12^*	2.00 × 10^−11^*
*Histological subtype*	Normal Adenocarcinoma	41	–		
	Mucinous	234	↑	1.63 × 10^−12 a^*	2.63 × 10^−01 a^*
	Adenocarcinoma	37	↑	4.80 × 10^−11 a^*	2.80 × 10^−12 a^*
*Nodal metastasis status*	Normal N_0_	41	–		
	N1	166	↑	1.00 × 10^−12^*^a^	1.00 × 10^−12^*^a^
	N2	70	↑	1.00 × 10^−12^*^ab^	1.00 × 10^−12^*^ab^
		47	↑	1.00 × 10^−12^*^b^	1.00 × 10^−12^*^b^

**Note**: -, normal expression; ↑, overexpression of both CDK-1 and CDK-4; non-cancer, normal people with no CRC within the set parameters.

**Fig. 5:**
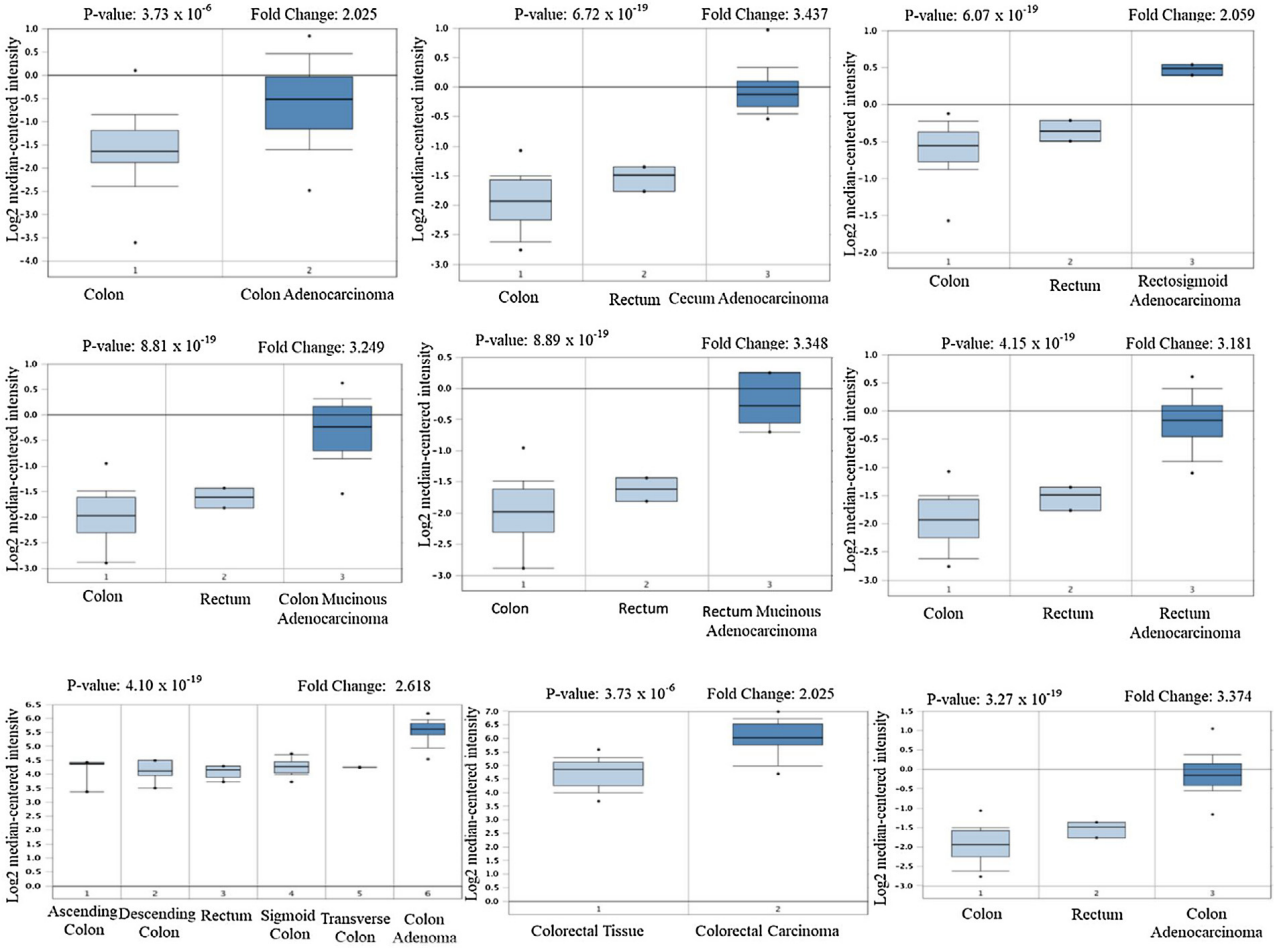
Analysis of CDK-4 gene expression in CRC. The Box plot was derived from gene expression data in the Oncomine database. The expression of specific CDK-4 in normal tissue vs cancer tissue.

### 3.3 Survival analysis

**Fig. 6:**
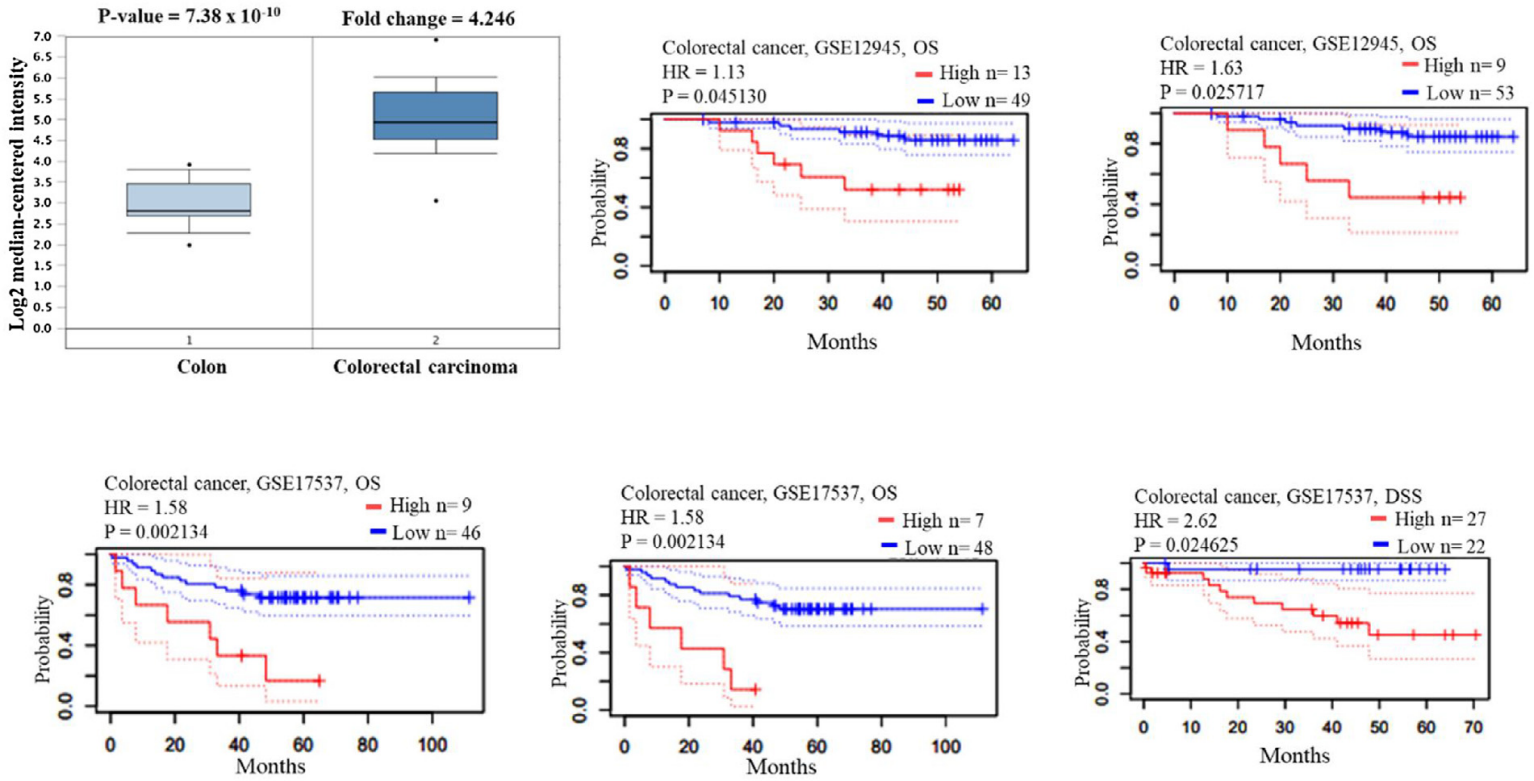
Gene expression and prognostic value of CDK-1 in CRC (Oncomine and Prognoscan databases).

**Fig. 7:**
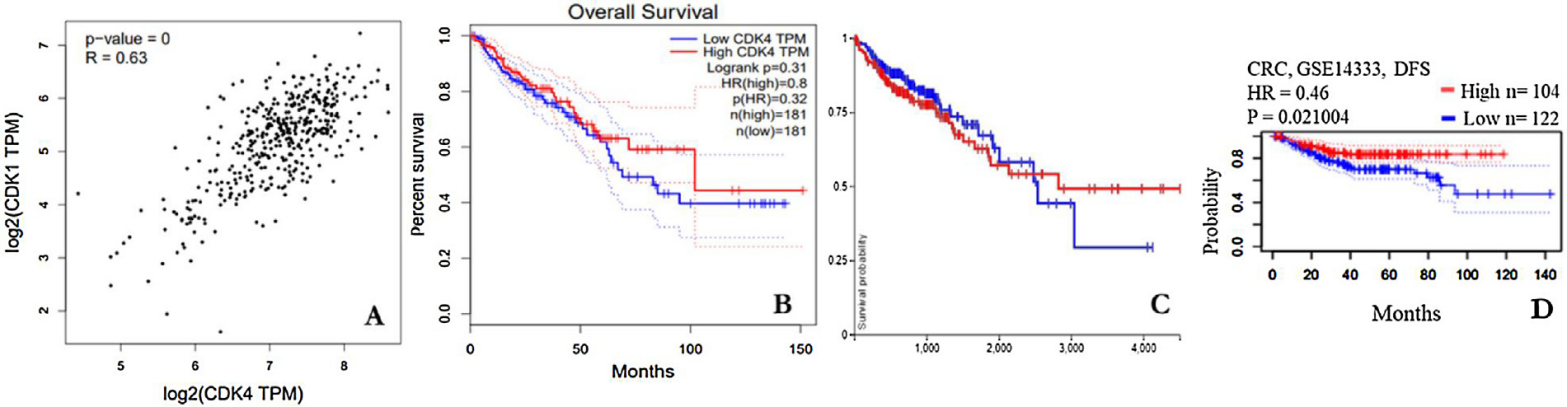
Correlation and Survival analysis of CDK-4 in CRC. (A) gene correlation between CDK-1 and CDK-4, Survival analysis of CDK-4 using (B) GEPIA database, (C) UCSC-Xena database and (D) PrognoScan database.

The prognostic values of CDK-1 and CDK-4 were assessed using the PrognoScan-based Kaplan-Meier analyses. The overall survival (OS) and disease-free survival (DFS) of CDK-1 were determined in five different datasets (*p* < 0.05) in CRC. The results obtained were consistent across the five datasets and confirmed that the expression of CDK-1 positively correlated with poor OS rates in CRC patients (Fig. 6). In contrast, when the prognostic value of CDK-4 was assessed in three different databases using the same bioinformatics tools (Fig. 7B-D), the expression of CDK-4 in the GSE14333 dataset inversely correlated to poor prognosis in disease-free survival (DFS) in CRC (Fig. 7). Although CDK-1 and CDK-4 were positively correlated, their prognostic values with respect to mRNA expression are different (Fig. 7A and Supplementary Fig. 1).

### 3.4 Protein partners of CDKs

**Fig. 8:**
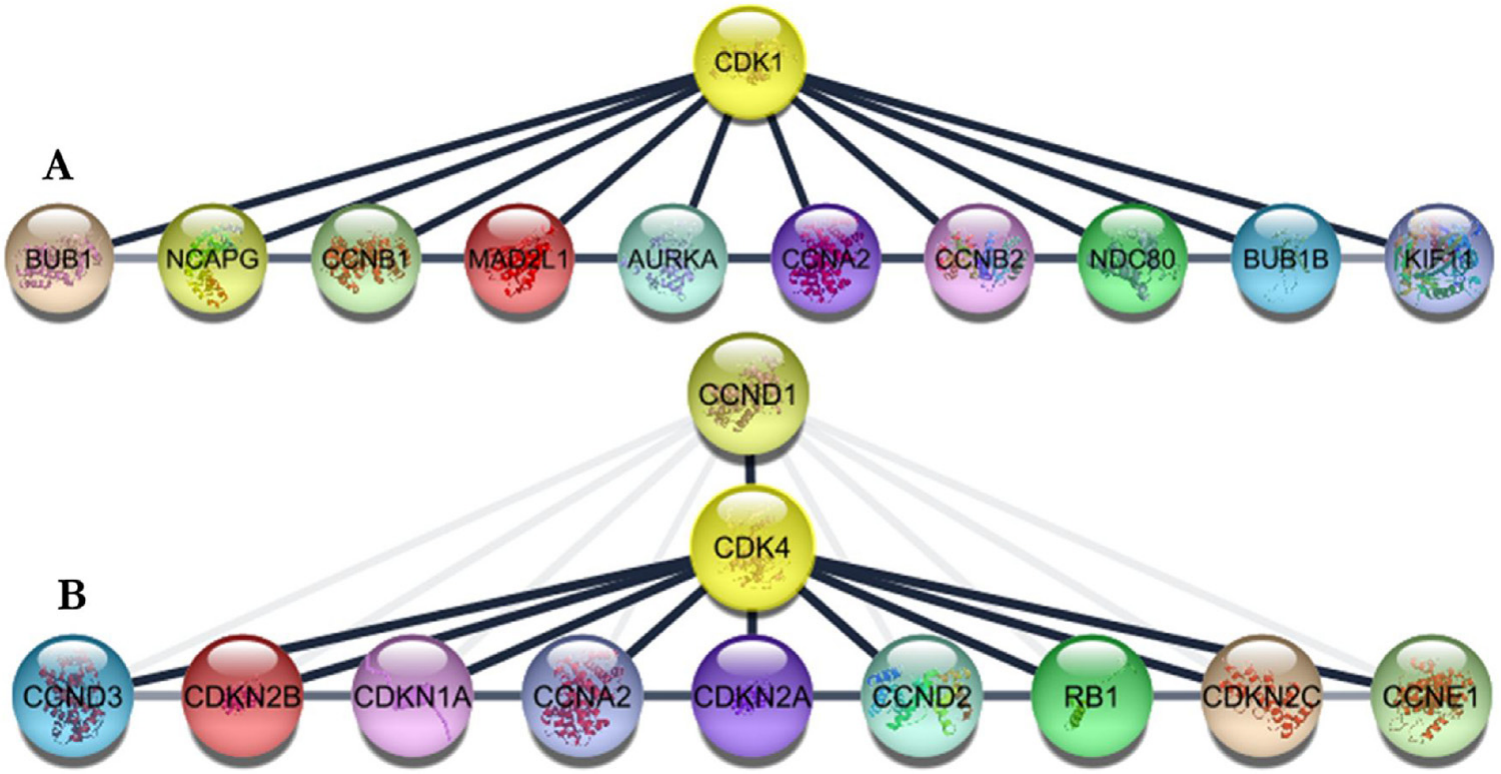
Identification of known and predicted structural proteins crucial for CDK function. Interacting nodes are displayed in colored circles (STRING v11 plugin Cytoscape 3.7.2). Predicted functional associations of CDK-1 (A) and CDK-4 (B) are shown based on experimental data, text-mining, co-expression, protein homology, and curated database entries. PPI enrichment *p*-value: 3.75 × 10^−14^.

Using CDK-1 and CDK-4 as a queries, associated proteins were generated using STRING v11 plugin Cytoscape 3.7.2with a 0.90 confidence level as the minimum required interaction. Each of these genes generated 10 other genes through curated database entries, experimental validation, text mining, co-expression and/or protein homology (Fig. 8).

### 3.5 Gene ontology of associated genes

To identify functional categories and characteristic biological attributes of CDKs (Fig. 8), Gene Oncology (GO) enrichment analysis was performed using the FunRich software.

**Fig. 9:**
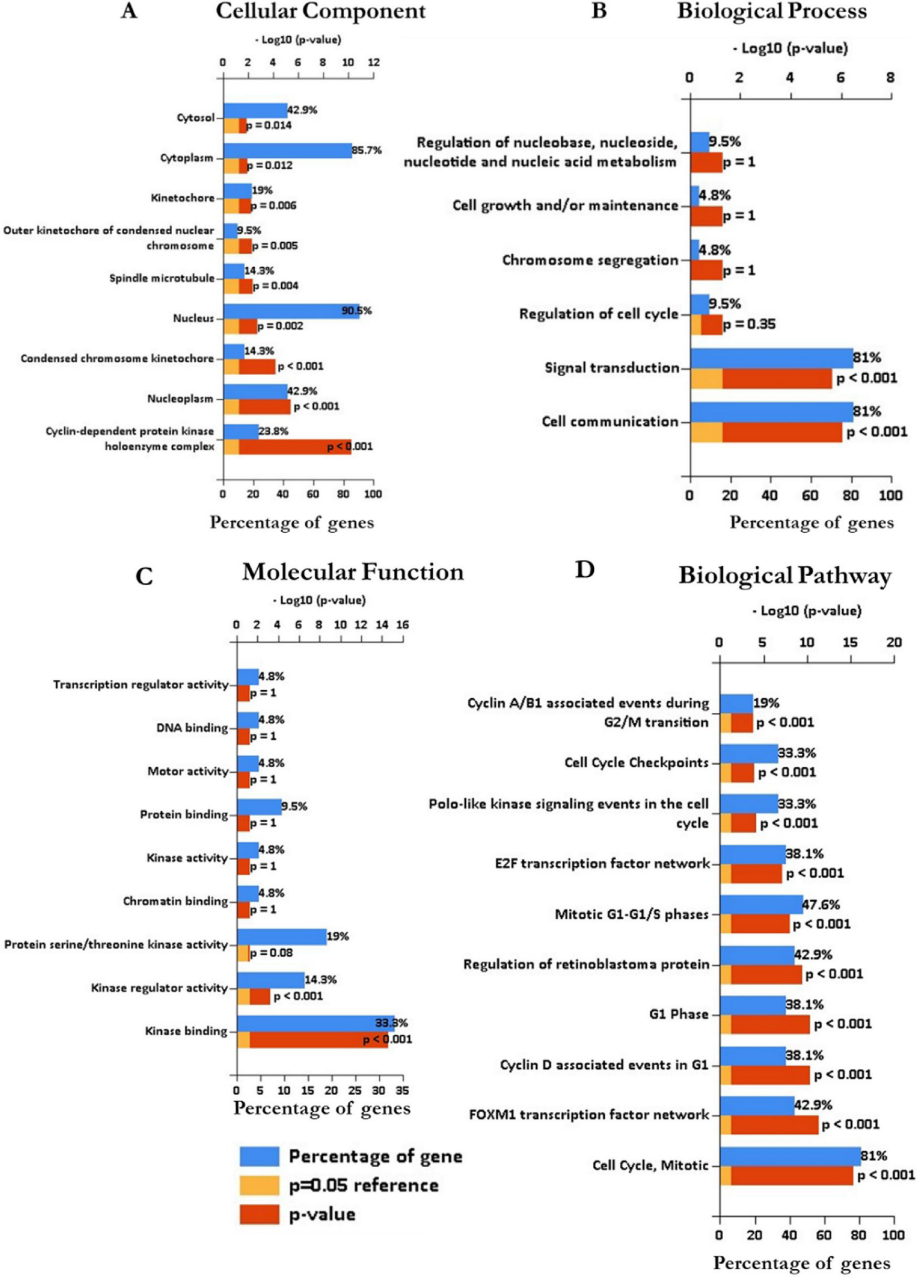
Gene Ontology annotation of significantly enriched associated proteins with CDKs showing (A) cellular component, (B) biological process, (C) molecular function and (D) biological pathway (FunRich software v3.1.3).

Analysis of GO terms were filtered for cellular component (CC), biological process (BP), molecular function (MF) and biological pathway (BP) (Fig. 9). The top 10 GO terms of the associated genes in BP, CC, MF and BP were considered significant at *p-*value of < 0.05 (Fig. 9A-D). These genes were involved in the different GO terms such as cell communication, ontology; BP, kinase binding, ontology; MF, cyclin-dependent protein kinase holoenzyme complex, ontology; CC and cell cycle, ontology; BP. The biological pathway analysis showed that cell cycle was highly enriched amongst the CDKs associated genes, which are closely related to FOXM1 transcription factor network, regulation of retinoblastoma protein and the E2F transcription factor network (Fig. 9D).

### 3.6 Genomic correlation of CDK genes in clinical data

**Fig. 10:**
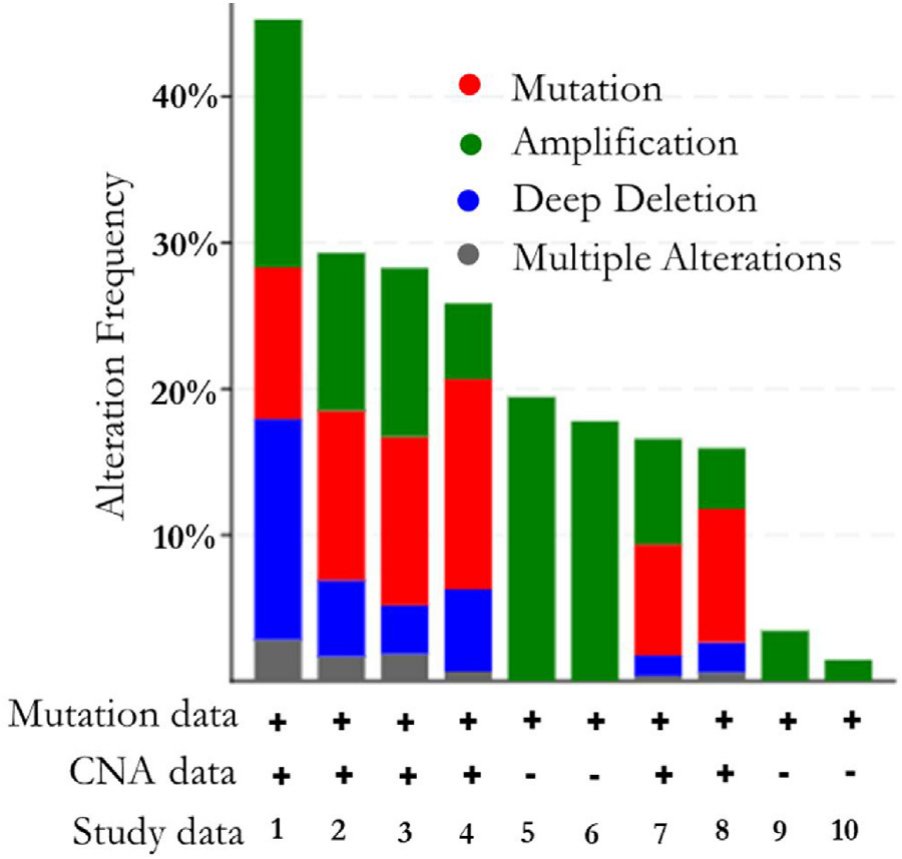
The alteration frequency of CDK-1 and CDK-4 along with the identified protein partners (A 21-gene) in 10 CRC studies. The studies include: COAD (CPTAC-2, 2019, Colorectal (TCGA, PanCan), Colorectal (TCGA, pub), Colorectal (TCGA), Colorectal (Genentech), Colorectal (DFCI, 2016), CRC (MSK, 2018), Rectal (MSK, 2019), COAD (Case CCC), and Colorectal (MSKCC) (cBioPortal).

**Fig. 11:**
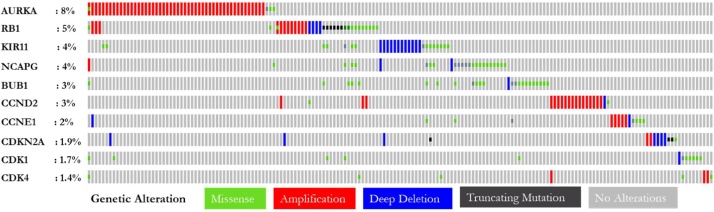
Alteration frequency of gene signatures in CRC. The Oncoprint feature (cBioPortal database) was used to identify the CNA frequency of each gene in CDK within two combined studies (CRC, TCGA, and COAD, CPTAC-2).

To lower the barriers of access to the complex data sets and accelerate the translation of genomic data into new biological insights, therapies, and clinical trials, the cBioPortal database was used to profile the CDK genes in ten CRC studies. This software tool accessed at https://www.cbioportal.org/ reduces molecular profiling data from cancer tissues and cell lines into readily understandable genetic, epigenetic, gene expression and proteomic events. The patterns of gene alterations were visualized in specific CRC studies and all the relevant genomic alterations in these studies were reported (Fig. 10, Fig. 11, Table 3, Table 4). The results are represented as a bar graph plot of alteration frequency (%) against mutation data, copy number alteration and study data (Fig. 10). The mutation observed after the query of the CDKs include mutation, amplification, deep deletion and multiple alteration. Table 3 shows the alteration frequency of CDKs in different CRC data sources. This table reported the percentages of occurrence in selected case number, in different types of alteration. In COAD (CPTAC-2, 2019) with a sample number of 105, the frequency of aleration observed were 17.14 %, 15.25 %, and 10.48 % for mutation, deep deletion and amplification respectively. Overall the highest form of alteration was observed for mutation in all the CRC cases except in Colorectal (TCGA) data where 14.62 % for amplification was observed as opposed 13.21 % for mutation.

**Table 3:** Alteration frequency of associated proteins signature with CDK-1 and CDK-4.

CRC Studies (Data source)	N	Freq. (%)	Amp% (N)	Mut.% (N)	Deep Del.% (N)	Mul. Alt. % (N)
COAD (CPTAC-2, 2019)	105	45.71	10.48 (11)	17.14 (18)	15.24 (16)	2.86 (3)
Colorectal (TCGA, PanCan)	526	30.01	10.65 (56)	12.17 (64)	5.32 (28)	1.90 (10)
Colorectal (TCGA, pub)	220	32.27	11.36 (25)	13.64 (30)	5.45 (12)	1.82 (4)
Colorectal (TCGA)	212	33.02	14.62 (31)	13.21 (28)	2.83 (6)	2.36 (5)
Colorectal (Genentech)	72	19.44	NA	19.44 (14)	NA	NA
Colorectal (DFCI, 2016)	619	17.77	NA	17.77 (110)	NA	NA
CRC (MSK, 2018)	1134	15.68	7.58 (86)	7.23 (18)	1.41 (16)	0.35 (4)
Rectal (MSK, 2019)	334	16.17	9.28 (14)	4.19 (14)	2.1 (7)	0.6 (2)
COAD (Case CCC)	29	3.45	NA	3.45 (1)	NA	NA
Colorectal (MSKCC)	138	1.45	NA	1.45 (2)	NA	NA

**Abbreviations:** N: study number (Cases); Freq: Frequency in Percentage; Mut %: Mutation; Deep Del%: Deep Deletion; Mul. Alt: Multiple Alterations; COAD (CPTAC-2, 2019): Colon Cancer (CPTAC-2 Prospective, Cell 2019); Colorectal (TCGA, PanCan): Colorectal Adenocarcinoma (TCGA PanCancer Atlas); Colorectal (TCGA, pub): Colorectal Adenocarcinoma (TCGA Firehose Legacy); Colorectal (TCGA): Colorectal Adenocarcinoma (TCGA, Nature 2012); Colorectal (Genentech): Colorectal Adenocarcinoma (Genentech, Nature 2012); Colorectal (DFCI, 2016): Colorectal Adenocarcinoma (DFCI, Cell Reports 2016); CRC (MSK, 2018): Metastatic Colorectal Cancer (MSKCC, Cancer Cell 2018); Rectal (MSK, 2019): Rectal Cancer (MSK Nature Medicine 2019); COAD (Case CCC): Colon Adenocarcinoma (CaseCCC, PNAS 2015); Colorectal (MSKCC): Colon Adenocarcinoma Triplets (MSKCC, Genome Biol 2014); and NA: Not Applicable.

**Table 4:** Genetic alteration summary of genes of interest in five CRC samples.

Data	CDK-1	CDK-4	CCNE1	CDKN2A	RB1	BUB1	NCAPG	AURKA	KIF11	CCND2
CRC, TCGA, Firehose	0.5 %	1.4 %	2..7 %	1.8 %	5%	3%	3%	8%	4%	2.7 %
CRC, TCGA, Nature	0.5 %	0.9 %	1.9 %	2.4 %	3%	2.8 %	3%	10 %	4%	1.9 %
CRC, Pan Cancer	1.9 %	1.1 %	1.5 %	2.1 %	5%	3%	4%	7%	2.9 %	3%
MSKCC	0%	0.7 %	1%	2.9 %	3%	0%	0%	4%	0%	1%
CPTAC-2	1.9 %	2.9 %	4%	1.9 %	7%	6%	6%	8%	11 %	2.9 %

Fig. 11 shows the percentages of alterations in CDK-4, CCNE1, CDKN2A, CCND2, RB1, CDK-1, BUB1, NCAPG, AURKA and KIF11 genes in the CRC. Grey bars along a vertical line represent the same sample interrogated for genetic alteration. With two combined studies containing 704 samples, 25 % of the queried genes were altered (178). The mutations observed in CRC were deep deletion, missenses and truncation mutations. Also, CRC may be thought to frequently amplify CDKs. With respect to the 10 querried genes, AURKA was frequently altered in CRC with 8% frequency of alteration with amplification as the major alteration followed by RB1 with 5% alterations (amplification, missense, deep deletion and truncating mutation). However, among there genes, CDKN2A, CDK1 and CDK4 were least altered with 1.9 %, 1.7 % and 1.4 % frequencies of alteration respectively. In addition, the percentages of alteration frequencies of five selected CRC samples (TCGA, Firehose; TCGA, Nature; Pan Cancer; MSKCC and CPTAC-2 were further investigated. The result shows that AURKA has the highest alteration frequency across all five samples which sugegest that the AURKA gene may be a good diagnostic biomarker for CRC (Table 4).

### 3.7 Gene hotspot mutation for CDKs

**Fig. 12:**
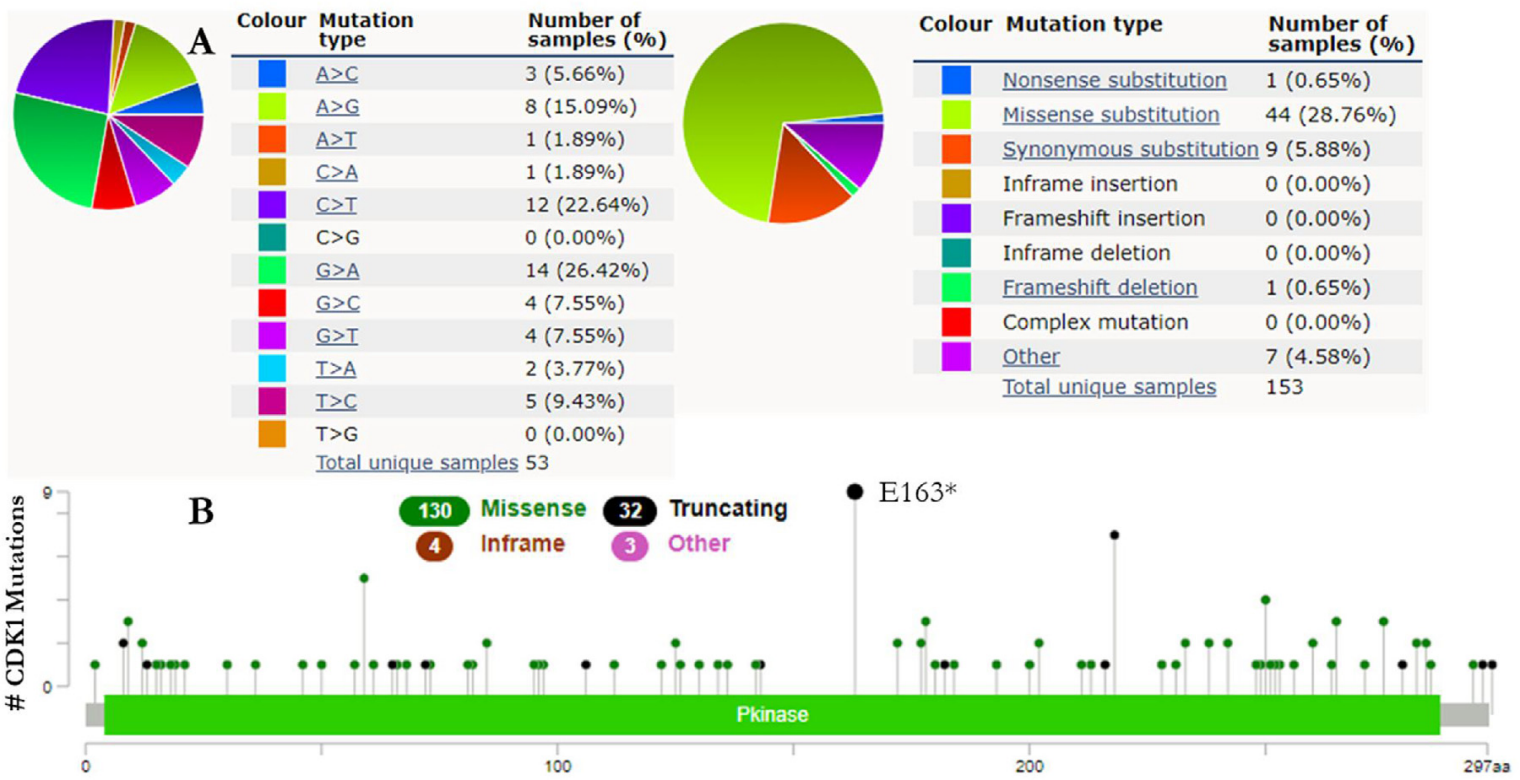
CDK-1 mutation in CRC patients. (A) The percentages of mutation types of CDK-1 in CRC were revealed in a pie chart generated from the COSMIC database. (B) cBioPortal was used to analyze the genetic alteration frequency of CDK-1 mutations in CRC.

**Fig. 13:**
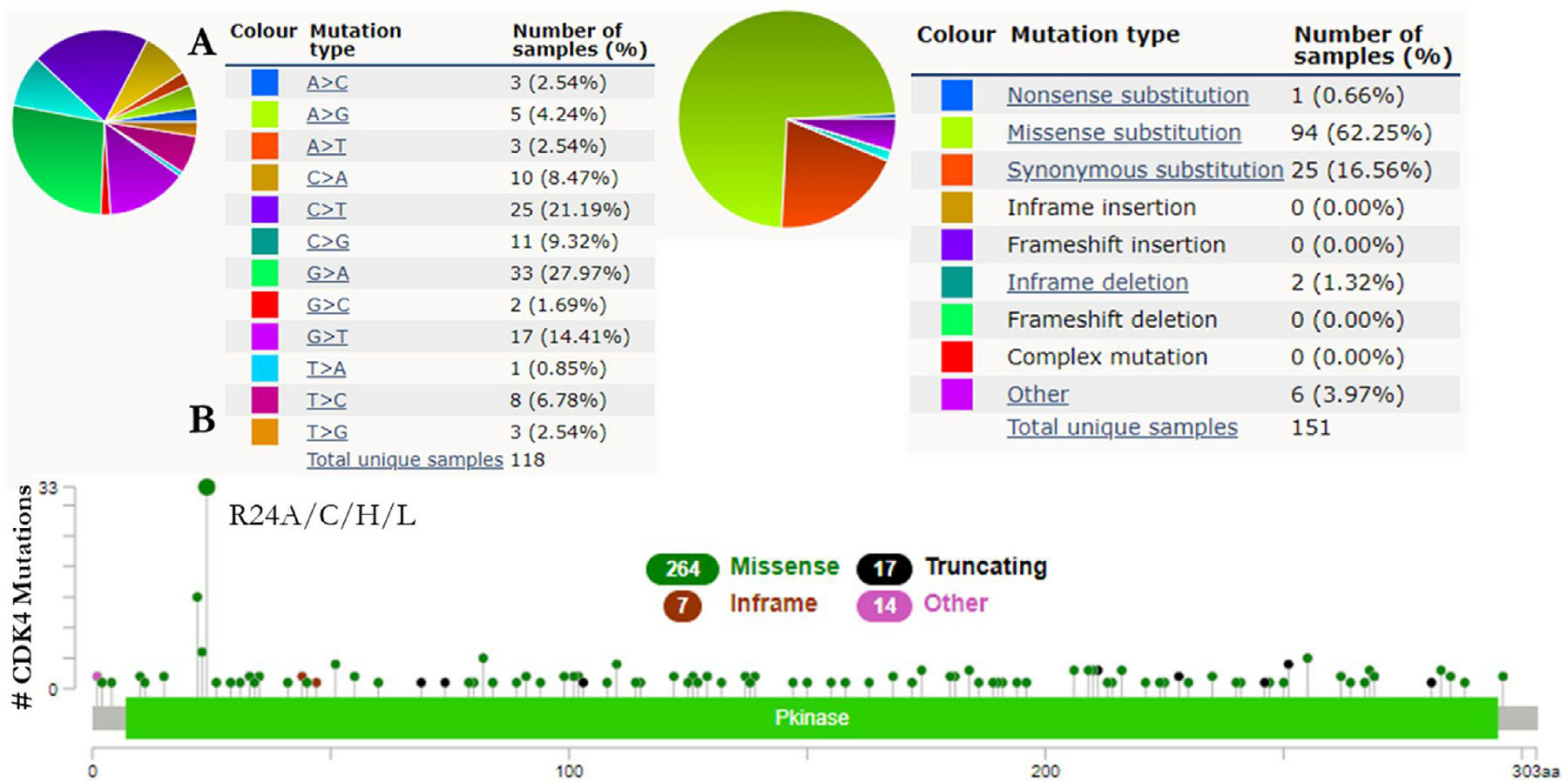
CDK-4 mutation in CRC patients. (A) The percentages of mutation types of CDK-4 in CRC were revealed in a pie chart generated from the COSMIC database. (B) cBioPortal was used to analyze the genetic alteration frequency of CDK-4 mutations in CRC.

The mutations of CDK-1 and CDK-4 were carried out using the COSMIC database for CRC coupled with the cBioPortal database for hotspot mutation in human cancers. These databases were queried for CDK-1 and CDK-4 gene mutations. The pie charts illustrate that the mutant types of CRC were primarily missense and synonymous substitutions (Figs. 12A and 13 A). The CDK-1 and CDK-4 CRC data contained G > A and C > T mutations, each accounting for over 20 % of the genes coding strand (Figs. 12A and 13 A). Using cBioPortal, 85,583 combined studies involving 82,346 patients from 277 studies of human cancers were investigated. CDK mutations across these studies reported a total of 169 and 302 mutations for CDK-1 and CDK-4 respectively. The mutation sites for CDK-1 was detected between 0 and 297 amino acids of the PKinase domain whereas the mutations site for CDK-4 was detected in the amino acids range of 0–303. The PKinase domain represent the hotspot domain for both genes (Figs. 12B and 13 B).

### 3.8 Immune infiltration analysis

**Fig. 14:**
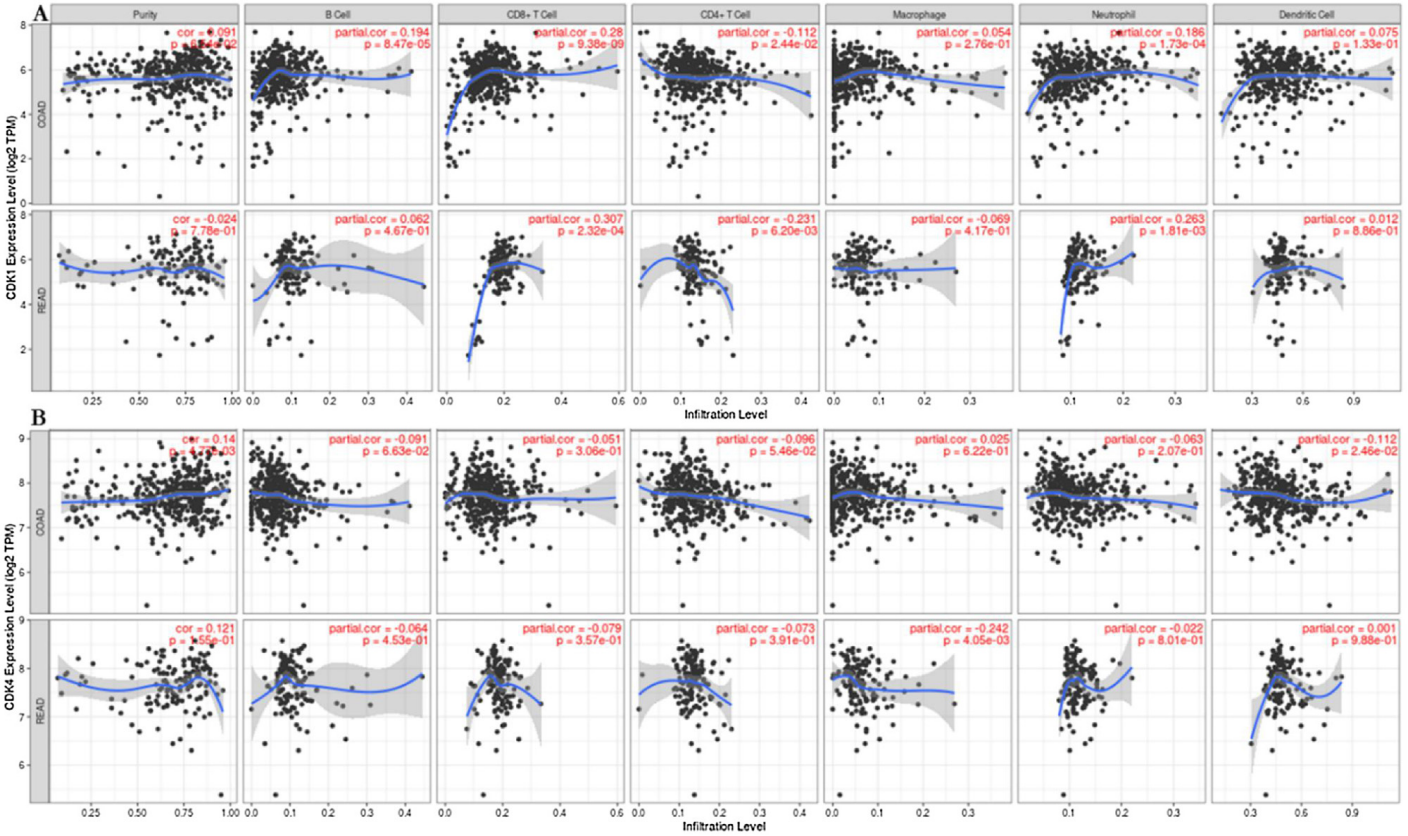
Correlation of CDK-1 and CDK4 expression with immune purity and fraction of immune cells in COAD (colon adenocarcinoma) and READ (rectum adenocarcinoma). The scatterplots showed the purity-corrected partial Spearman's correlation and statistical significance. (A) CDK-1 expression is significantly positively related to tumor purity as well as B cells, CD8 + T cells, macrophages, neutrophils, and dendritic cells and has significant negative correlations with infiltrating level of CD4 + T cells in COAD (n = 457). In contrast, a negative correlation was observed in tumor purity as well as CD4 + T and macrophages in READ. (B) CDK-4 expression has significant correlations with tumor purity and infiltrating levels of macrophages in COAD and dendritic cells in READ (n = 166).

TIMER was used to investigate the molecular characterization of tumor-immune cell interaction. The genes of interest were used as queries against the six immune infiltrates using two cancer types (COAD and READ). The output of each query generated scatter plots for each of the infiltrates in COAD and READ (Fig. 14). In COAD, CDK-1 and CDK-4 clearly shows significant positive correlations with tumor purity while in READ, only CDK-4 was positively correlated with tumor purity. The expression levels of each gene were displayed against tumor purity (left). This is because genes that are highly expressed in the microenvironment are expected to have negative association with tumor purity. For each immune cell, the partial Spearman’s correlation was adjusted by tumor purity (Fig. 14).

## 4 Discussion

CDKs participate in the regulation of cell cycle progression and transcription, and aberrant expression of these genes is often observed in various human tumors. Dysregulation in cell cycle progression has been associated with bowel (small and large intestine) diseases particularly colon and rectum cancers representing one-fifth of all cancers globally [57]. Unlike normal cells, cancer cells grow uncontrollably without regulation by cell-cycle due to a loss of function of checkpoint integrity. This could be due to CDK inhibitor inactivation leading to overexpression of either cyclin or CDKs. Therefore, these kinases are potential targets for cancer theranostics. Using bioinformatics approaches, this study investigated the roles of CDKs in relation to their expressions and immune infilterates in CRC. Most adenocarcinomas of the colorectum arise in a visible benign precursor lesion, the adenoma, which is a monoclonal proliferation of dysplastic nonmalignant epithelial cells [58]. Therefore, both adenoma and adenocarcinoma of the colorectum were considered in this study.

The expression of CDKs was explored in 20 different cancer subtypes with emphasis on different sections of the colorectum, using different computational tools. Oncomine, TIMER and GEPIA web-based tools were used to evaluate and validate the mRNA expression of CDK-1 and CDK-4 in different cancer types (solid tumor and hematological malignancies), different CRC sections as well as clinico-pathological parameters of CRC in relation to normal tissues. Relative to normal colorectum tissue, gene expression of CDK-1 and CDK-4 were up-regulated in all major forms of cancer with high mRNA expressions observed in CRC. Xue et al. [59] revealed that the expression of CDK-1 was exceptionally high in CRC in contrast with normal tissues, and it predicts distance metastasis risk in CRC; CDK-1 can also alter the progression of CRC through phosphorylation of JAK1 to initiate the JAK/STAT3 signaling pathway. In addition, high expression of CDK-1 reportedly stimulate the proliferation and migration of CRC while its inhibition restricted CRC proliferation [60]. Furthermore, frequent CDK-1 expression was correlated with therapeutic target, and the therapeutic resistance of BRAF mutant human CRC can be suppressed by targeting CDK-1 [3]. Similarly, poor prognosis was observed in patients with high expression of CDK-4 (*P* < 0.001) [61]. Therefore, inhibition of these kinases may be useful in cancer therapy and their expression patterns could become a novel prognostic marker in CRC.

The protein expression level was also explored for reliable prognostic values for CRC patients using *in silico* web-based servers. Bioinformatics analyses have provided useful results based on large experimentally validated data accesible in various databases. Gene expression prognostic signature for high-grade serous ovarian cancer was investigated by resampling/cross-validation method with Cox regression analysis followed by Kaplan–Meier analysis and log-rank testing [62]. Similarly, He et al. [63] probed the survival analysis of the expression level of MANF in HCC using the Kaplan–Meier method. In addition, Cox regression models were used to ascertain the prognostic value. Following this pattern, the prognostic values of CDK-1 and CDK-4 were assessed using the comprehensive survival analysis platform PrognoScan-based Kaplan-Meier analyses. Expression of CDK-1 positively correlated with poor OS and DFS in CRC patients while the CDK-4 expression level of this study inversely correlated with poor prognosis in DFS. Although CDK-1 and CDK-4 were positively correlated, their prognostic values with respect to mRNA expression are contrasting. According to Li et al. [64] the overexpression of CDK-1 in patients with CRC was associated with decreased OS. Moreover, high mRNA expression of CDK-1 is linked to poor OS in CRC, hepatocellular carcinoma and lung cancer [[65], [66], [67]]. This result revealed that CDK-1 expression levels in CRC correlated with the poor OS of patients with the disease. In addition, high expression of CDK-1 is a prognostic factor for CRC.

Protein associations have been previously reported to be pivotal in biological processes and cellular function in both prokaryotes and eukaryotes including humans (normal and disease states) [68]. The discovery of associated proteins with specific gene(s) of interest are important to understand the mode of action of diseases such as cancer to improve the development of therapeutic drugs and treatmentsstrategies. Numerous experimental approaches have been deployed for the identification of interacting genes but the processes are demanding with limited outputs. However, bioinformatics approach has been increasingly used to validate and predict elusive protein partners. Therefore, this study identified twenty associated proteins with CDK-1 and CDK-4 using STRING v11 plugin Cytoscape 3.7.2 since proteins are constantly regulated and rarely function in isolation. These genes are crucial to the roles and specific molecular networks of CDK-1 and CDK-4 in diseases, most importantly CRC.

The ten associated proteins identified for CDK-1 include Budding Uninhibited By Benzimidazoles 1 (BUB1), Non-SMC Condensin I Complex Subunit G (NCAPG), Cyclin B1 (CCNB1), Mitotic Arrest Deficient 2 Like 1 (MAD2L1), Aurora Kinase A (AURKA), Cyclin A2 (CCNA2), Cyclin B2 (CCNB2), NDC80 Kinetochore Complex Component (NDC80), BUB1 Mitotic Checkpoint Serine/Threonine Kinase B (BUB1B), and Kinesin Family Member 11 (KIF11) with protein-protein interaction (PPI) enrichment *p*-value of 3.75 × 10^−14^, average node degree of 9.27 and average local clustering coefficient of 0.943.

With an enrichment *p*-value of 4.44 × 10^−16^, average node degree of 10 and average local clustering coefficient of 1.0, CDK-4 was equally associated with ten genes namely; Cyclin D3 (CCND3), Cyclin-Dependent Kinase Inhibitor 2B (CDKN2B), Cyclin-Dependent Kinase Inhibitor 1A (CDKN1A), Cyclin-Dependent Kinase Inhibitor 2A (CDKN2A), Cyclin D2 (CCND2), Cyclin D1 (CCND1), RB Transcriptional Corepressor 1 (RB1), Cyclin-Dependent Kinase Inhibitor 2C (CDKN2C), Cyclin E1 (CCNE1), and Cyclin A2 (CCNA2). A few lines of research have reported the expressions of these genes in different cancers [[68], [69], [70], [71]]. Mizuarai et al. [69] reported the *in vivo* and *in vitro* expression of CCND1/CDKN2A status in 30 cancer cell lines composed of 16 RB1-positive and 14 RB1-negative cancers. The study identified a panel of genes which could predict RB1 status in both cultured cancer cell lines and clinical tumor samples. AURKA genes was studied to provide cancer therapeutic interventions due to their significant expression in cancer patient samples [70]. Similarly, Korobeynikov et al. [71] suggested that the dual targeting of AURKA and PAK1 may be a promising therapeutic strategy for cancer treatment from a combinatory study of alisertib and FRAX1036. Another study by Ding et al. [72] shows that MAD2L1 may be a novel oncogene in an *in vitro* 20 clinical CRC samples. Since previous studies have predicted the association of these genes as promising targets in cancer therapy [73,74], CDKs together with their protein partners could serve as a confirmation of the strength of the methodology used in this study.

The discovery of pathways and specific processes that are significant with factors regulating activities to genes of interest is important in cancer research. FunRich software was used to evaluate the enrichment of the protein partners in BP, MF and CC. These genes were involved in: a) cell communication and signal transduction in BP, b) kinase binding and kinase regulator activity in MF, c) cyclin-dependent protein kinase holoenzyme complex and nucleoplasm in CC and d) cell cycle, mitotic and FOXM1 transcription factor network in BP. These processes can be further probed for their involvement in CRC in order to tailor specific strategies for CRC therapeutics.

In a combined study of 3,953 samples, the associated genes were altered in 21 % (817) of queried samples reporting 46 % altered cases as the alteration frequency (cancer types summary). From the bar chart, the alterations include mutation, amplification, deep deletion and multiple alterations. The mutation data and copy number alterations were also represented. The ratio of alteration ranged between 1.45 and 45.71 % with the dominance hierarchy as Colon Cancer (CPTAC-2 Prospective, Cell 2019), Colorectal Adenocarcinoma (TCGA, Nature 2012) and Colorectal Adenocarcinoma (TCGA Firehose Legacy). Furthermore, alterations in the associated genes (CDK-1, CDK-4, CCNE1, CDKN2A, RBI, NCAPG, AURKA, and CCND2) were determined in five CRC studies (CRC, TCGA, Firehose, CRC, TCGA, Nature, CRC, Pan-Cancer, MSKCC and CPTAC-2). The percentages of alterations in these genes ranged from 0 to 11% for individual genes. AURKA and RB1 genes were amplified predominantly in the combined studies, suggesting that their expression could be significant to CRC prognosis. Since these genes are positively correlated with CDK genes, their prognostic values deserve to be assessed for theranostic purposes.

The alterations frequencies and hotspot mutations of CDK-1 and CDK-4 were analyzed with COSMIC and cBioPortal databases. The results revealed that the types of mutations of CDK-1 and CDK-4 in the CRC data were missense mutations among others. Furthermore, the alteration frequency of these genes in CRC is notably low. CDK genes were altered in 2,537 of the queried samples (3%), with a somatic mutation frequency of 0.2 % for both CDK-1 and CDK-4. For CDK-1, 169 mutations including 130 missenses, 32 truncating, 4 in-frame insertions, and 3 others with E163* hotspot were observed. In a total of 302 mutations for CDK-4, including 264 missenses, 17 truncating, 7 in-frame insertions, and 14 others its hotspot was identified as a R24A/C/H/L hotspot. The clinical implications of these mutation hotspots are likely to be oncogenic with gain of function as the biological effect. CDK-1 and CDK-4 are significant kinases in M-phase andG1 stages of cell-cycle respectively. Unlike the aberrant expression of CDK-1 in stage-2 colon and rectum adenocarcinoma being associated with a higher risk of metastasis [75], mutations in the catalytic subunit of CDK-4 gene has also been reported to be common and its abnormal expression has been uncovered in over 90 % of all bowel cancers [76]. The identified hotspots for these kinases may be a promising drug target.

During tumor development and progression, cancer and immune cells interact by multiple genes and pathways. Therefore, it is worthy to explore the correlation between gene expression and immune infiltration level. Tumor purity plays an important role in understanding the pathogenic mechanism of tumors [77]. This is a crucial characteristic that cannot be ignored in cancer genomics or epigenomics data analysis [78]. It is noteworthy that the increase in tumor purity was inversely correlated with the expression of CDK-1 in READ. Specifically, the immune infiltration analysis may have resulted from an increase in the numbers of infiltrating B cells, CD8+cells, neutrophils, and dendritic cells. Conversely, an increase in tumor purity was positively correlated with the expression of CDK-1 in COAD due to CD4+cells and CDK-4 in COAD and READ resulting from a fraction of immune cells.

## 5 Conclusion

Dysregulation of kinase genes is commonly encountered in bowel cancers. In this study, CDK-1 and CDK-4 genes were overexpressed in all the CRC subtypes with respect to normal tissues and the expression of CDK-1 was associated with poor prognosis as opposed to CDK-4. Based on the associated proteins, RB1 and AURKA were positively correlated and showed significant relationships with CDKs and their expressions were significantly altered in CRC. The most frequent alteration was found to be a mutation with hotspots E163* and R24A located on the Pkinase domain for both CDK-1 and CDK-4 respectively. In addition, one of the crucial findings of the current study was the identification of a positive correlation between CDK-1 and increased numbers of infiltrating B cells, CD8+ cells, neutrophils and dendritic cells and a contrasting correlation between CDK-4 and the immune infiltrates in both CRC subtypes. This is a potential novel feature of CRC in the context of the immune response, driven by CDK-1 and CDK-4. In concluson, the results of this study justified exploring CDKs as prognostic markers as well as immunotherapeutic target for CRC as well as other cancers. The role of these genes in CRC and the potential molecular mechanisms deserve further molecular investigation.

## Data availability

The datasets generated during and/or analysed during the current study are presented in the manuscript. All other databases used for data generation are duly referenced and their links are also provided.

## Ethical approval and consent to participate

Not applicable.

## Consent for publication

Not applicable.

## Author contributions

All authors have made significant contributions to the submission of this manuscript. A.O.F, N.R.S.S, A.K, AM, and M.M conceived the concept of the work. A.O.F, AK, AM, and MM designed the study. A.O.F, O.O.B, A.K, A.M and M.M carried out the research, data analyses and supervision. Manuscript draft by A.O.F, N.R.S.S, and O.O.B. M.M, A.K, and N.R.S.S critically appraised the manuscript. All authors agreed with the submission of the final version of the article.

## Funding

Not applicable.

## Declaration of Competing Interest

The authors report no declarations of interest.
