# Supplementary material for: Expression of cyclin-dependent kinases and their clinical significance with immune infiltrates could predict prognosis in colorectal cancer
Source: Biotechnol Rep (Amst). 2021 Feb 23;29:e00602. doi: 10.1016/j.btre.2021.e00602 (PMC7937668; doi:10.1016/j.btre.2021.e00602)
Supplement: Supplementary file 1 [file mmc1.docx]

**Expression of Cyclin-Dependent Kinases and their Clinical Significance with immune infiltrates could predict prognosis in Colorectal Cancer**

Adewale Oluwaseun Fadaka^*1^ Nicole Remaliah Samantha Sibuyi^1^ Olalekan Olanrewaju Bakare^2^, Ashwil Klein^3^, Abram Madimabe Madiehe^1,4^and Mervin Meyer^1^

^1^Department of Science and Innovation/Mintek Nanotechnology Innovation Centre, Biolabels Node, Department of Biotechnology, Faculty of Natural Sciences, University of the Western Cape, Bellville, South Africa.

^2^Bioinformatics research group, Department of Biotechnology, Faculty of Natural Sciences, University of the Western Cape, Private Bag X17, Bellville, 7535 Cape Town, South Africa.

^3^Plant Omics group, Department of Biotechnology, Faculty of Natural Sciences, University of the Western Cape, Private Bag X17, Bellville, 7535 Cape Town, South Africa.

^4^Nanobiotechnology Research Group, Department of Biotechnology, Faculty of Natural Sciences, University of the Western Cape, Bellville, South Africa.

*** Correspondence:** [**afadaka@uwc.ac.za**](mailto:afadaka@uwc.ac.za)

**Supplementary Figure**


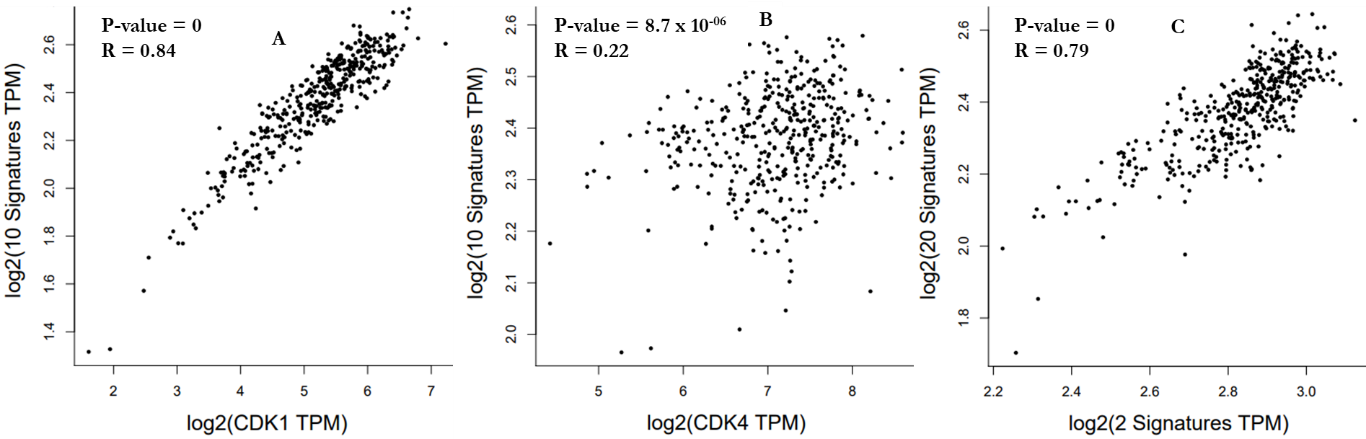


**Supplementary Figure 1:** Co-expressed genes validation (GEPIA). (a) Positive correction of (a) CDK-1 and associated genes (b) CDK-4 and associated genes (c) signatures of associated genes and genes of interest. TPM, transcripts per million.
